# Supplementary material for: Neuronal Hyperactivity Induces Tau Phosphorylation Selectively in Excitatory Pyramidal Neurons Which Contributes to GluA2 Regulation
Source: bioRxiv. 2026 Oct 28:2026.10.26.762028. Preprint. [Version 1] doi: 10.64898/2026.10.26.762028 (PMC13733932; doi:10.64898/2026.10.26.762028)
Supplement: Supplementary file 1 [file media-1.docx]

**Supplemental Material**

**Neuronal Hyperactivity Induces Tau Phosphorylation Selectively in Excitatory Pyramidal Neurons Which Contributes to GluA2 Regulation**

Manyi Sun^1^, Kevin Vaughey^1^, Bian Liu^2,3^, Haidan Guo^1^, Richard Huganir^2,3^, Meaghan Morris^1,3^

^1^Department of Pathology, Johns Hopkins School of Medicine, Baltimore, MD, 21205.

^2^Department of Neuroscience, Johns Hopkins School of Medicine, Baltimore, MD, 21205.

^3^Kavli Neuroscience Discovery Institute, Baltimore, MD 21205.

**Supplemental Figure 1: Tau Phosphorylation with Neuronal Activity Changes.** Rat hippocampal neurons were treated with TTX or bicuculline for 24 hours. (A) Western blot showing subtle increased tau phosphorylation (pS202/T205 normalized to total tau) with bicuculline treatment with (B) quantification. (C) Total tau levels (normalized to tubulin) did not change. (D) Representative image showing no increase in phospho-tau (green) in inhibitory interneurons (GAD67, magenta) with bicuculline treatment. Bic, bicuculline; RU, relative units; TTX, tetrodotoxin; Tub, tubulin; Veh, vehicle. Scale bar is 25 µm.

**Table S1: Kinase Inhibitor Screen**

| **Inhibitor** | **Target** | **Concentration** | **Dendritic pTau Effect** |
| --- | --- | --- | --- |
| Lithium chloride | GSK3β | 2 mM | No inhibition |
| GO6983 | PKC | 100 nM | No inhibition |
| (S)-Roscotivine | CDK5 | 20 µM | No inhibition |
| IC261 | CK1 | 20 µM | No inhibition |
| TBB | CK2 | 50 µM | No inhibition |
| SB203580 | P38 MAPK | 20 µM | No inhibition |
| Ly294002 | PI3K | 20 µM | No inhibition |
| U0126 | MEK1/2 | 10 µM | No inhibition |
| H89 | PKA | 5,10 µM | Inhibited |
| KT5720 | PKA | 3 µM | Inhibited |
